# Surgical management of laparoscopic cholecystectomy (LC) related major bile duct injuries; predictors of short-and long-term outcomes in a tertiary Egyptian center- a retrospective cohort study

**DOI:** 10.1016/j.amsu.2018.11.006

**Published:** 2018-11-16

**Authors:** Emad Hamdy Gad, Eslam Ayoup, Yasmin Kamel, Talat Zakareya, Mohamed Abbasy, Ali Nada, Mohamed Housseni, Mohammed Al-sayed Abd-elsamee

**Affiliations:** a Hepatobiliary Surgery, National Liver Institute, Menoufia University, Shebein Elkoum, Egypt; b Anaesthesia, National Liver Institute, Menoufia University, Shebein Elkoum, Egypt; c Hepatology and Endoscopy, National Liver Institute, Menoufia University, Shebein Elkoum, Egypt; d Radioligy, National Liver Institute, Menoufia University, Shebein Elkoum, Egypt

**Keywords:** Laparoscopic cholecystectomy, Biliary injury, Liver cirrhosis, Sepsis, Outcome of BI repair

## Abstract

**Objectives:**

Laparoscopic cholecystectomy - associated bile duct injury is a clinical problem with bad outcome. The study aimed to analyze the outcome of surgical management of these injuries.

**Patients and methods:**

We retrospectively analyzed 69 patients underwent surgical management of laparoscopic cholecystectomy related major bile duct injuries in the period from the beginning of 2013 to the beginning of 2018.

**Results:**

Regarding injury type; the Leaking, Obstructing, leaking + obstructing, leaking + vascular, and obstructing + vascular injuries were 43.5%, 27.5%, 18.8%, 2.9%, and 7.2% respectively. However, the Strasberg classification of injury was as follow E1 = 25, E2 = 32, E3 = 8, and E4 = 4. The definitive procedures were as follow: end to end biliary anastomosis with stenting, hepaticojejunostomy (HJ) with or without stenting, and RT hepatectomy plus biliary reconstruction with stenting in 4.3%, 87%, and 8.7% of patients respectively. According to the time of definitive procedure from injury; the immediate (before 72 h), intermediate (between 72 h and 1.5months), and late (after1.5 months) management were 13%, 14.5%, and 72.5% respectively. The hospital and/or 1month (early) morbidity after definitive treatment was 21.7%, while, the late biliary morbidity was 17.4% and the overall mortality was 2.9%, on the other hand, the late biliary morbidity-free survival was 79.7%. On univariate analysis, the following factors were significant predictors of early morbidity; Sepsis at referral, higher Strasberg grade, associated vascular injury, right hepatectomy with biliary reconstruction as a definitive procedure, intra-operative bleeding with blood transfusion, liver cirrhosis, and longer operative times and hospital stays. However, the following factors were significantly associated with late biliary morbidity: Sepsis at referral, end to end anastomosis with stenting, reconstruction without stenting, liver cirrhosis, operative bleeding, and early morbidity.

**Conclusion:**

Sepsis at referral, liver cirrhosis, and operative bleeding were significantly associated with both early and late morbidities after definitive management of laparoscopic cholecystectomy related major bile duct injuries, so it is crucial to avoid these catastrophes when doing those major procedures.

## Highlights

• Sepsis at referral was associated with poor outcome after management of LC related MBDIs.
• Liver cirrhosis and operative bleeding were associated with poor outcome after management of these injuries.
• It is crucial to avoid these catastrophes when doing those major procedures.

## List of abbreviations:

ACC: Acute calcular cholecystitis
ASA: American society of anesthesia
BDI: Bile duct injury
BI: Biliary injury
CBD: Common bile duct
CDS: Clavien Dindo score
CT: computerized tomography
CUSA: Cavitron ultrasonic surgical aspirator
ERCP: Endoscopic retrograde cholangio-pancreatography
HA: Hepatic artery
HJ: Hepaticojejunostomy
HPB: Hepatopancreatobiliary
IOC: Intra-operative cholangiogram
IRB: Institutional review board
KM: Kaplan–Meier
LC: Laparoscopic cholecystectomy
LFT: Liver function test
MBDIs: Major bile duct injuries
MRCP: Magnetic resonance cholangiopancreatography
MRI: Magnetic resonance imaging
NLI: National Liver Institute
OC: Open cholecystectomy
PDS: polidioxanone
POD: Post-operative day
PTC: Percutaneous transhepatic cholangiography
PTD: Percutaneous transhepatic drainage
PV: Portal vein
RUQ: Right upper quadrant
US: Ultrasonography

## 1 Introduction

Despite increased surgical skills and experience regarding laparoscopic choelcystectomy (LC), the rate of LC related bile duct injury (BDI) is still higher in comparison to open cholecystectomy (0.2%–1.5% vs. 0.1–0.2% respectively) [[1], [2], [3], [4], [5], [6], [7], [8]]. LC related BDIs range from minor injuries to complex hilar injuries as classified by Strasberg et al. [9,10]; where the major types correspond to type E injuries including ongoing stricture, complete occlusion, resection or division of the bile ducts; [[11], [12], [13]]. There are several risk factors for the occurrence of these injuries (I.e. Surgeon inexperience, misinterpretation of biliary anatomy, poor visualization of the surgical field, inflammation, Mirrizi's syndrome, excessive fibrosis in Calot's triangle, adhesions, hemorrhage and lack of intra-operative cholangiogram (IOC) [2,[14], [15], [16], [17], [18]].

The mechanisms of these injuries involve thermal injuries, scissors, ligatures or clips [15,16,[18], [19], [20]]. These injuries are dangerous with significant morbidity, and mortality [13,[21], [22], [23], [24], [25]]. Moreover, failure or delay in the early recognition or inappropriate management of them leads to catastrophic consequences [26,27].

Surgical biliary-enteric reconstruction including the Hepp-Couinaud approach at a specialist hepatobiliary center by an experienced surgeon, is the most effective treatment of these injuries with perfect long-term results [3,20,[28], [29], [30], [31]]. However, end-to-end biliary anastomosis can be utilized as a treatment strategy if major BDI (MBDI) is detected during surgery, with no extensive tissue loss, or inflammation [32]. Nevertheless, symptomatic patients with associated vascular lesions, lobar parenchyma atrophy, or abscesses benefit from hepatectomy [2,[33], [34], [35]].

There is little literature on the long-term outcomes after surgical reconstruction of MBDIs, moreover, the factors predicting those outcomes have not been studied extensively [14,30,[36], [37], [38]]. Our study aimed to analyze the early and late outcomes of surgical management of LC- related MBDIs in a tertiary referral center.

### 1.1 Patients and methods

Ninety patients underwent definitive surgical management of LC related MBDIs (>E1), in the period from the beginning of 2013 to the beginning of 2018 either in the department of hepato-pancreato-biliary (HPB) surgery (tertiary referral center), National Liver Institute (NLI), University of Menoufia, Menoufia, Egypt (88 patients), or in the calling hospitals in Menoufia (2 patients). After exclusion of cases with data loss and cases that refused research; our study included 69 patients (68 patients referred from other hospitals including the 2 patients that underwent the definitive repair in the calling hospital by our team and then referred), and one patient who had the injury in our hospital). After approval of institutional review board (IRB), we did this cohort study which is a single-institution retrospective analysis of a prospectively collected database that analyzed the outcome of surgical management of LC related MBDI in the period from mid 2017 to mid 2018, where patients were observed from POD1 until the end of June 2018 or until death of patients with median follow up period of 43 ms, range (0.7–66 ms), with researchregistry2211.

The data were collected from our records in our HPB surgery department including the data of the 3 patients operated in the calling hospitals. Written informed consents regarding surgery and researches were obtained from our patients. Our work has been reported in line with the STROCSS criteria [39].

The recorded data included patient demographics, indications of cholecystectomy, IOC performance during cholecystectomy, direct cause of biliary injury (BI) and timing of its discovery, referral time after injury, presentation of patients and presence of sepsis at referral, tools of biliary anatomy determination and BI classification, associated vascular injury, intervention before definitive operation and preoperative American society of anesthesia (ASA) score, time of definitive procedure from injury and type of operations, intra-operative liver biopsy results, operative bleeding and time, patients outcome, and lastly follow-up data.

Patient presentation ((i.e Jaundice, cholangitis, bilious drains, biliary peritonitis) was known by good history, clinical examination, and laboratory investigations especially liver function tests (LFTs))), furthermore, sepsis at referral was known by leukocytosis, abdominal pain, fever, and/or peritonitis [13]. However, cholangitis at referral was determined by the presence of leukocytosis, fever, and hyperbilirubinemia >3 mg/dL [40]. The patients with biliary peritonitis, cholangitis or sepsis were managed initially by intravenous antibiotics, aggressive nutritional management, biliary drainage (Endoscopic retrograde cholangiography (ERCP) or percutaneous trans-hepatic drainage (PTD)), and percutaneous or surgical drainage of abdominal collections; then definitive repair of these patients was delayed until control of sepsis.

**Fig. 1:**
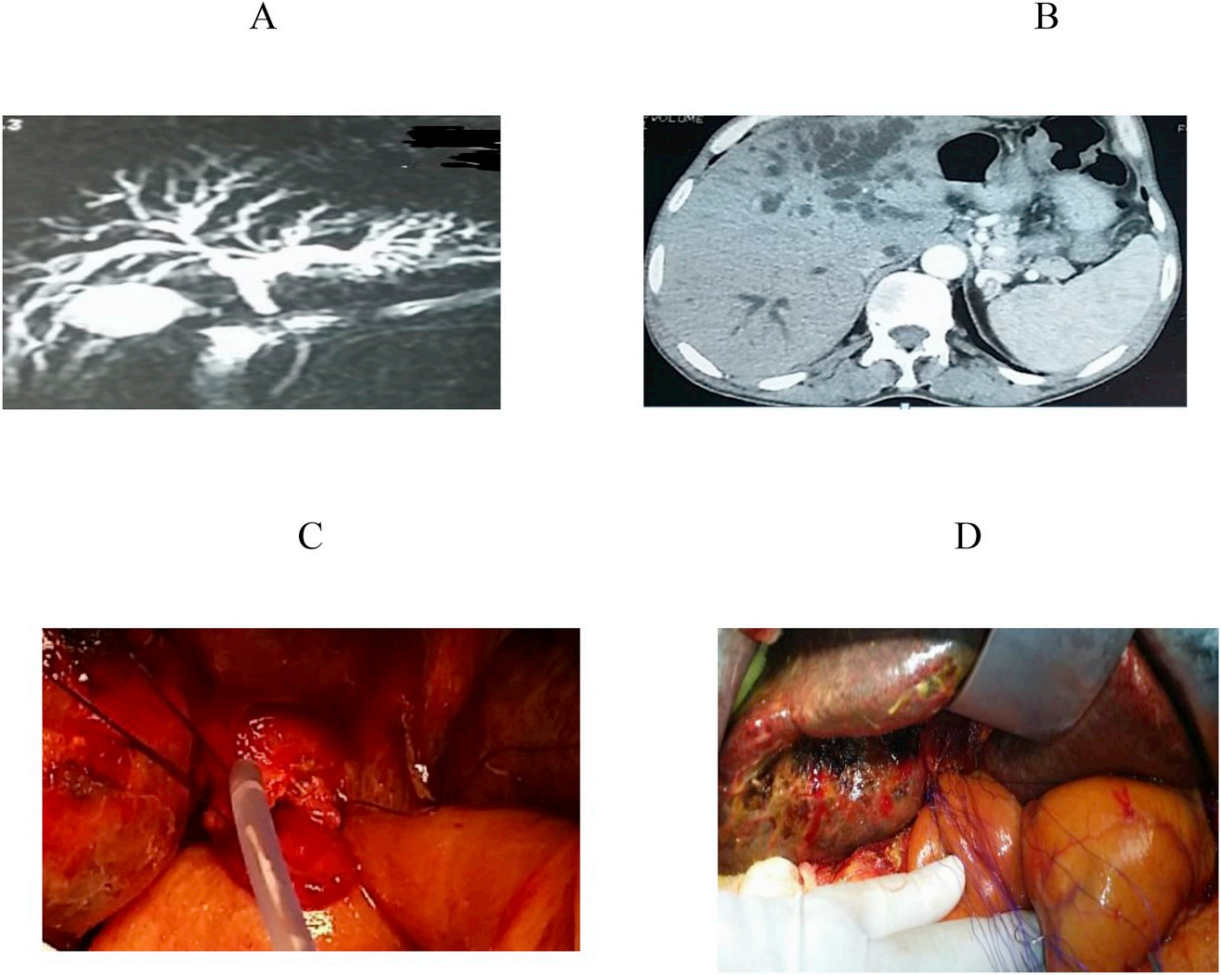
A, B: MRCP and CT showing type E1 BI, C, D: RY HJ without stent.

**Fig. 2:**
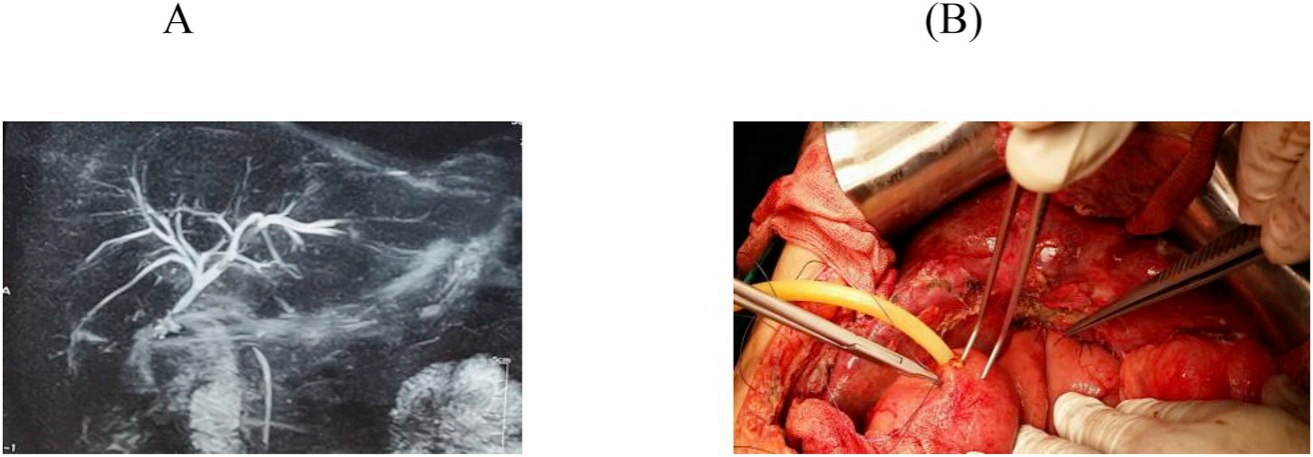
A: MRCP showing BI type E1, B: HJ with stent.

**Fig. 3:**
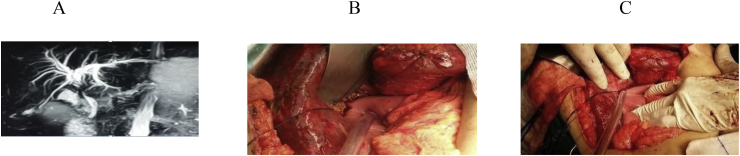
A- MRCP showing Type E3 biliary stricture, B,C: HJ without stent steps.

**Fig. 4:**
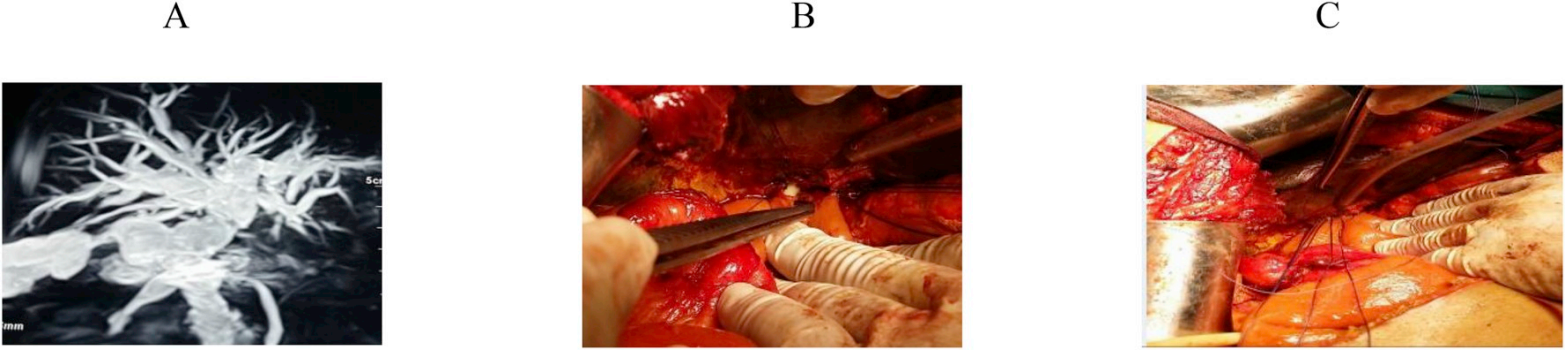
A: MRCP showing BI typeE3, B,C, HJ with stent.

**Fig. 5:**
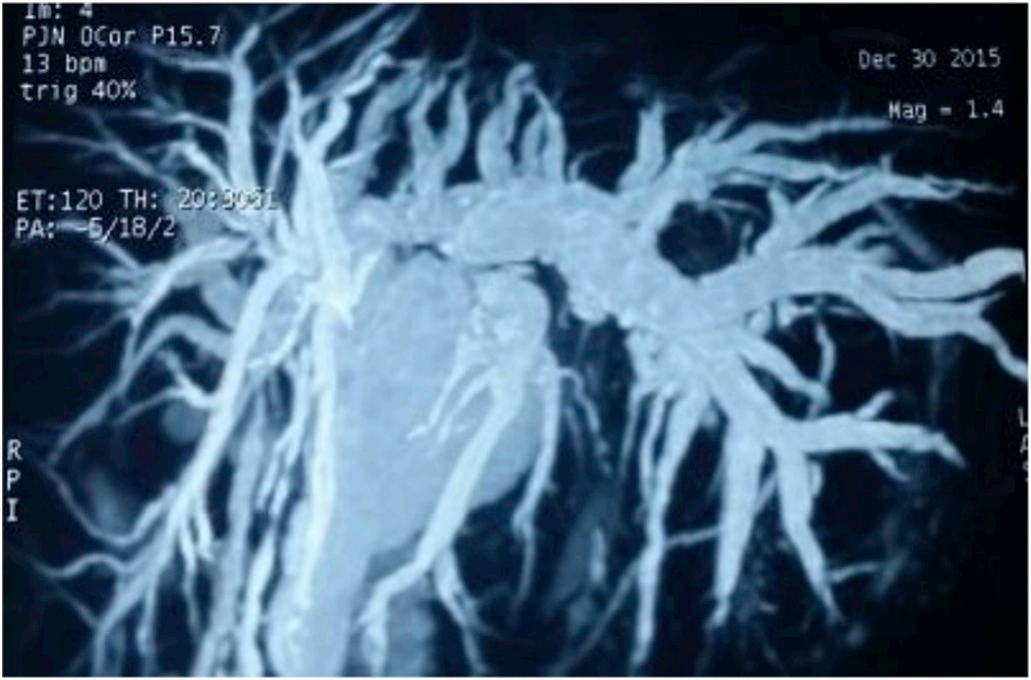
MRCP showing type E4 injury with RT lobe atrophy underwent RT hepatectomy with biliary reconstruction.

**Fig. 6:**
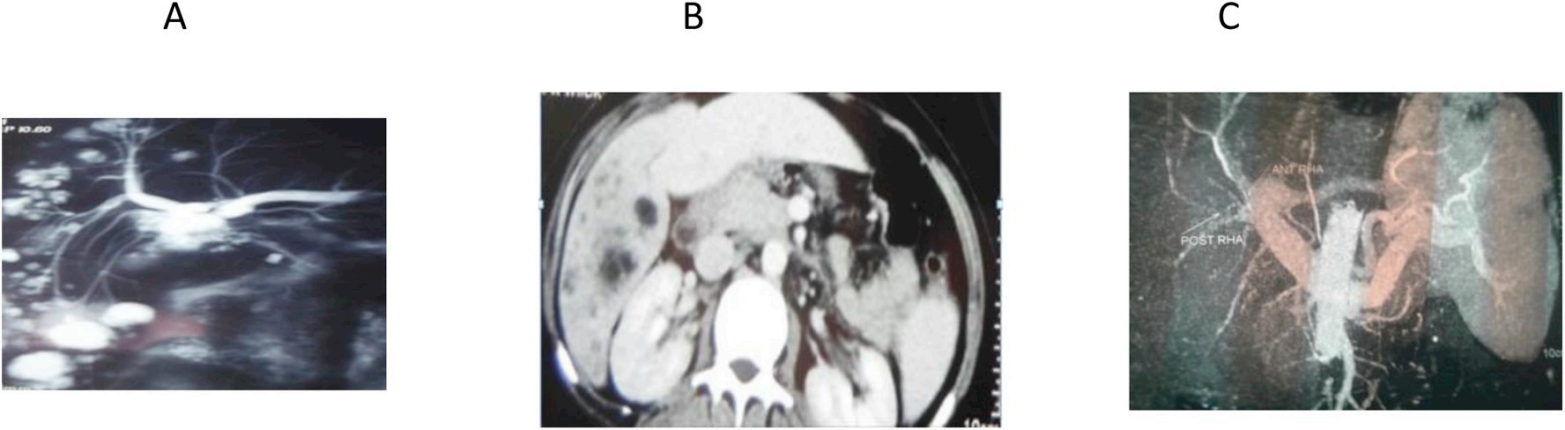
A,B,C: MRCP, CT and CT angiography respectively showing BI type E4 with RT HA and PV injuries and multiple hepatic abscesses in RT lobe, this patient underwent RT hepatectomy with biliary reconstruction.

For determination of biliary anatomy, abdominal US was done for all referred patients, abdominal CT, Magnetic resonance cholangio-pancreatography(MRCP) and fistulogram were done as indicated, however CT angiography was done when co-vascular injury was suspected, lastly ERCP and percutaneous trans-hepatic cholangiography/drainage (PTC)/PTD were done for diagnostic and therapeutic purposes. Fig. 1, Fig. 2, Fig. 3, Fig. 4, Fig. 5, Fig. 6.

The anatomic extent of BI was classified according to the Strasberg- Bismuth classification system [9,41] Fig. 1, Fig. 2, Fig. 3, Fig. 4, Fig. 5, Fig. 6; moreover, BI was classified into leaking, obstructing, both leaking and obstructing and lastly BI associated with vascular injury.

After referral to our center, multidisciplinary staff meeting occurred including surgeons, endoscopists, radiologists, and anaesthetists for controlling initial patient condition where different intervention procedures were performed before the definitive operation (I.e.laparotomy, endoscopic and/or radiologic ones); the laparotomy included drainage of biliary collection ± external biliary diversion, where the endoscopy included ERCP ± sphinectrotomy ± dilatation ± stenting and lastly, the radiology included percutaneous pigtail drainge ± PTD.(N.B. some of these procedures were done as definitive management of certain types of BI but failed, furthermore, the procedures were done by the authors of the manuscript according to their specialities)).

The time of performance of operation from injury was classified into immediate (during the 1st 72 h), intermediate (between 72 h and 1.5 months) and late (after1.5 months).

Operation type: Our institutional guidelines is performing HJ with or without trans-anastomotic stent (Hepp-Couinaud approach) (Fig. 1, Fig. 2, Fig. 3, Fig. 4), however, with higher biliary injuries associated with vascular injuries, liver abscesses and/or atrophy, hepatectomy with biliary reconstruction with trans-anastomotic stenting was our choice, lastly, end to end biliary anastomosis with internal stent was our procedure to keep the normal biliary anatomy, keep the sphinecter of Oddi function, and make a chance for future ERCP if MBDI was detected during index surgery, with no extensive tissue loss, or inflammation. (N.B stent means tube drain I.e. T-tube, nelatone tube, straight biliary stent or ureteric catheter).

The surgical techniques: After opening the abdomen, adhesiolysis and mobilization of the liver were done. Intraoperative doppler-ultrasound was used to detect vascular involvement (if suspected), Pringle maneuver was systematically done, hilar structures were exposed and IOC was performed to assess the biliary tree [20].

In HJ cases, the anastomosis was performed with a Roux-en-Y HJ, with extension to the left hepatic duct (Hepp– Couinaud approach) [42] to achieve adequate stoma size where absorbable sutures were used with or without trans-anastomotic stenting depending upon the stricture type, and technical difficulty [3,19,20,30,37,43]. However, in RT hepatectomy cases, the parenchymal transection was performed using cavitron ultrasonic surgical aspirator (CUSA) in combination with the harmonic scalpel and then a Roux-en-Y HJ between the LT hepatic duct and the jejunum using trans-anastomotic stenting was performed. Lastly, end to end biliary anastomosis was done by interrupted absorbable (polidioxanone) PDS 5/0 using internal stents.

Stents were internal or external; the internal stents were 4–7 French straight biliary stent (9–11 cm) or it was 4–7 French ureteric catheters that were placed into the common bile duct (CBD) and blindly directed across the ampulla of Vater. However, the external stent tip was positioned into the bile duct mostly the left one crossing the HJ anastomosis and pulled out of the jejunum [4,44]. They were removed 1.5–3 months from surgery after doing cholangiogram (ERCP or tube cholangiogram) and ensuring the absence of stricture or leak, Liver biopsy was obtained during operation to determine the presence of 2ry biliary cirrhosis, moreover, operative bleeding, blood transfusion and operative time were recorded.

The outcome of patients: It was classified into 1- Early (hospital and/or 1month) morbidities that occurred during the initial hospital stay or after discharge until the 1st month after surgery and classified according to Clavien-Dindo grading system [45]. 2-Early mortalities, 3- Late biliary morbidities that occurred after 30 days from surgery until the end of follow-up period ((graded according to McDonald et al., 1995 [46]; Grades A (Asymptomatic with normal LFT), B (Asymptomatic with mild LFT changes and/or occasional episodes of fever or pain), C (Cholangitis and abnormal LFT), and D (PTD or surgical revision)); grades A and B were considered success however; grades C and D were considered failure, 4- Late mortalities.

Long-term follow-up: For detection of late biliary morbidity, patients were followed-up every 3 months in the 1st year then yearly until the end of follow-up period by clinical assessment, LFT, ultrasonography, and others if needed (i.e. MRCP).

Statistical Techniques: Th data were processed with SPSS software (Statistical Product and Service Solutions, version 21, SSPS Inc, Chicago, IL, USA). Nonnumerical data were expressed in frequency and % and analyzed with the ^Qui square^ or Fisher exact tests. Numerical data were expressed as the mean and standard deviation and were compared with the T or Mann whitteny tests. Univariate analysis and then multivariate analysis (by Binary logistic regression method) were done to detect the relationship between the different pre- and intra-operative variables and early morbidity as well as the relation between these variables and late biliary morbidity. The Kaplan–Meier method was used for survival analysis to assess the overall and late biliary morbidity-free survivals, a P value of <0.05 was significant.

## 2 Results

### 2.1 The characteristics of patients

They were classified as 31(44.9%) males, and 38 (55.1%) females; their mean age was 38.08 ± 9.6. The previous cholecystectomy was done due to acute calcular cholecystitis (ACC) and biliary colic in 66.7% and 33.3% of them respectively; furthermore, IOC was done in 15.9% of patients during LC. Clipping, diathermy, ligature, and scissors were the direct cause of injury in 17.4%, 62.3%, 8.7% and 11.6% of them respectively. BI was discovered during cholecystectomy in 20.3% of patients, on the other hand, it was diagnosed in the early (during 7 days from cholecystectomy), intermediate (7 days–3 months) and late (after 3 months) periods after cholecystectomy in 44.9%, 26.1%, and 8.7% of them respectively. Furthermore, those patients with BI discovery after cholecystectomy were mainly presented with jaundice, cholangitis, bilious drain, and peritonitis that affected 23.2%, 17.4%, 30.4%, and 8.7% of them respectively. The mean time of referral to our center after injury diagnosis was7.1 ± 8.9 days, however, 10 (14.5%) of patients had sepsis at referral; this sepsis was due to biliary peritonitis or severe cholangitis with or without cholangectitic abscesses. The abdominal US, CT abdomen, abdominal CT angiography, MRCP, PTC, and ERCP were done in 65(94.2%), 14 (20.3%), 11 (15.9%), 40(58%), 10(14.5%), and 35(50.6%) of them respectively.

According to the nature of biliary injury, it was classified into Leaking, obstructing, leaking + obstructing, leaking + vascular, and obstructing + vascular that affected 43.5%, 27.5%, 18.8%, 2.9%, and 7.2% of patients respectively. On the other hand and regarding Strasberg classification of injury, the E1, E2, E3, and E4 types affected 36.2%, 46.4%, 11.6% and 5.8% of them respectively. In our biliary injured patients, the associated vascular injury was as follow: RT hepatic artery (HA) injury, RT HA + RT portal vein(PV) injuries, and RT PV injury that affected 5(7.2%), 1(1.4%), and 1(1.4%) of our patients respectively. Table 1.

**Table 1:** Characteristics of patients.

Character	NO	(%)
	69	(100%)
	(Mean ± SD)	
Age(years) (Mean ± SD)	38.08 ± 9.6	
Gender		
Males	31	(44.9%)
Females	38	(55.1%)
Indications of cholecystectomy		
ACC	46	(66.7%)
Biliary colic	23	(33.3%)
Co morbidity	13	(18.8%)
IOC during cholecystectomy	11	(15.9%)
Direct cause of injury		
Clipping	12	(17.4%)
Diathermy	43	(62.3%)
Ligature	6	(8.7%)
scissor	8	(11.6%)
Time of injury diagnosis		
During cholecystectomy	14	(20.3%)
Early(during 7 days from cholecystectomy)	31	(44.9%)
Intermediate(7 days to 3 months)	18	(26.1%)
Late (after 3 months)	6	(8.7%)
Main presentation		
Discovery during **cholecystectomy**	14	(20.3%)
Jaundice	16	(23.2%)
Cholangitis	12	(17.4%)
bile from drain	21	(30.4%)
Peritonitis	6	(8.7%)
Referral pattern		
Referred before definitive repair	65	(94.3%)
Referred after definitive repair(done by our team after calling us)	3	(4.3%)
Our center injury(No referral)	1	(1.4%)
Sepsis at referral	10	(14.5%)
Referral time after injury diagnosis (days) (Mean ± SD)	7.1 ± 8.9	
Imaging		
US	65	(94.2%)
CT abdomen	14	(20.3%)
CT angiography	11	(15.9%)
MRCP	40	(58%)
PTC	10	(14.5%)
ERCP	35	(50.6%)
BI type		
Leaking	30	(43.5%)
Obstructing	19	(27.5%)
Both leaking and obstructing	13	(18.8%)
Leaking and vascular	2	(2.9%)
Obstructing and vascular	5	(7.2%)
Strasberg classification of injury		
E1	25	(36.2%)
E2	32	(46.4%)
E3	8	(11.6%)
E4	4	(5.8%)
Associated vascular injury		
RTHA injury	5	(7.2%)
RTHA, RTPV injury	1	(1.4%)
RTPV injury	1	(1.4%)

ACC: Acute calcular cholecystitis, IOC: Intra operative cholangiogram, US: Ultrasonography, CT: computerized tomography, MRCP: Magnetic resonance cholangiopancreatography, PTC: Percutaneous transhepatic cholangiography, ERCP: Endoscopic resonance cholangiopancreatography, BI: Biliary injury, RT HA: Right hepatic artery, RT PV: Right portal vein.

### 2.2 Management of injury

Fifty-six (81.2%) of patients underwent intervention procedures before the definitive operation in the form of laparotomy, endoscopy and/or intervention radiology; laparotomy was done in 21 patients where 16 of them underwent exploration for leak and drainage, and the other 5 patients underwent external biliary diversion, however, endoscopic intervention was done in 35 ones where 25 of them underwent ERCP and stenting, 7 patients underwent ERCP but failed dilatation or stenting, and the other 3 ones had cannulation failure, on the other hand, 26 patients underwent intervention radiology where 16 of them underwent pigtail drainage(single or multiple), 9 patients underwent PTD, and the last one underwent both pigtail drainage and PTD. Patients were graded as regard ASA score into 55(79.7%), 10(14.5%), and 4(5.8%), ASA 1, 2, and 3 respectively. The mean pre definitive procedure total and direct bilirubin were 6.5 ± 6.3 and 5.1 ± 4.9 (mg/dl) respectively.

The mean time of definitive procedure from injury was 74.5 ± 61.8 days (range, 0–210 days) where 9 (13%), 10 (14.5%), and 50(72.5%) of patients underwent the definitive operation immediately (during the 1st 72 h), in the intermediate period (between 72 h and 1.5 months) and in the late period (after1.5 months) from injury respectively. The definitive procedures were as follow: End to end direct biliary anastomosis with stenting in 3 patients, HJ without stent in 20 patients, HJ with stent in 40 patients, and hepatectomy with biliary reconstruction with stenting in 6 patients. Regarding the 3 patients who underwent end to end anastomosis with stent; the 1st one was due to our center injury that was discovered during LC, so conversion to open surgery was decided and after performing IOC; anastomosis was done using PDS5/0 interrupted sutures with internal 4 French straight biliary stent (9 cm), however, the other 2 patients had the injury in other centers where we were called and did the repair immediately in these centers using PDS 5/0 interrupted sutures with internal 7 French ureteric catheters. On the other hand, regarding the 6 patients who underwent hepatectomy; the 1st one had E3 injury and associated RT PV injury and liver atrophy, the 2nd one had E4 injury and associated RT HA injury, RT PV injury and liver abscesses, the 3rd one had E4 injury and associated RT HA injury and liver abscesses, while the last 3 ones had associated RT HA injury and liver atrophy (2 of them had E4 and one had E3 injuries). Thirteen (18.8%) and 10(14.5%) of patients had cirrhotic liver and operative bleeding during the definitive operation respectively. Lastly, the mean blood transfusion and operative time after the definitive procedure were 0.2 ± 0.7 units, and 227.8 ± 85.5 min respectively. Table 2.

**Table 2:** Management of injury.

Character	NO	(%)
	69	(100%)
	(Mean ± SD)	
Intervention before definitive treatment	56	(81.2%)
Laparotomy	21	(30.4%)
Endoscopic	35	(50.7%)
Radiologic	26	(37.7%)
ASA score		
1	55	(79.7%)
2	10	(14.5%)
3	4	(5.8%)
Total bilirubin(mg/dl)(Mean ± SD)	6.5 ± 6.3	
Direct bilirubin(mg/dl)(Mean ± SD)	5.1 ± 4.9	
Time of definitive procedure from injury		
Immediate (during the 1st 72 h)	9	(13%)
Intermediate (between 72 h and 1.5 months)	10	(14.5%)
Late (after1.5 months)	50	(72.5%)
Time of definitive procedure from injury(days) (Mean ± SD) (rang)	(74.5 ± 61.8)	(0–210)
Definitive procedure		
End to end biliary anastomosis with stent	3	(4.3%)
HJ with stent	40	(58%)
RT hepatectomy, HJ with stent	6	(8.7%)
HJ **without stent**	20	(29%)
Intra operative liver biopsy		
Cirrhotic	13	(18.8%)
Normal	56	(81.2%)
Operative bleeding	10	(14.5%)
Blood transfusion(units) (Mean ± SD)	0.2 ± 0.7	
Operative time(min) (Mean ± SD)	227.8 ± 85.5	

ASA: American society of anesthesia, HJ: Hepaticojejunostomy.

### 2.3 Outcome of patients after the definitive procedure and its predictors

The early (hospital and/or 1month) morbidity affected 15(21.7%) of patients (N.B some of the patients had more than one complication) where, early infection (pulmonary and/or wound infection), abdominal collection, bile leak, and cholangitis affected 10(14.5%), 4(5.8%), 4(5.8%), and 4(5.8%) of them respectively. However, these complications were graded regarding Clavien grading (CDS) as 6(8.7%), 6(8.7%), 1(1.4%) and 2(2.9%) grades 2, 3, 4, and 5 respectively (N.B in patients with multiple complications, we recorded only the highest CDS). Furthermore, the early mortality was 2.9% of patients (2 patients); the 1st one had associated vascular injury (HA + PV injuries) and liver atrophy and underwent RT hepatectomy + biliary reconstruction with stenting but unfortunately died 24 days after operation from liver failure and sepsis, however the other one had associated HA injury with multiple liver abscesses and underwent RT hepatectomy + biliary reconstruction with stenting and died 20 days from surgery due to liver failure and sepsis too. The mean hospital stay was 8.6 ± 5.2 days.

**Fig. 7:**
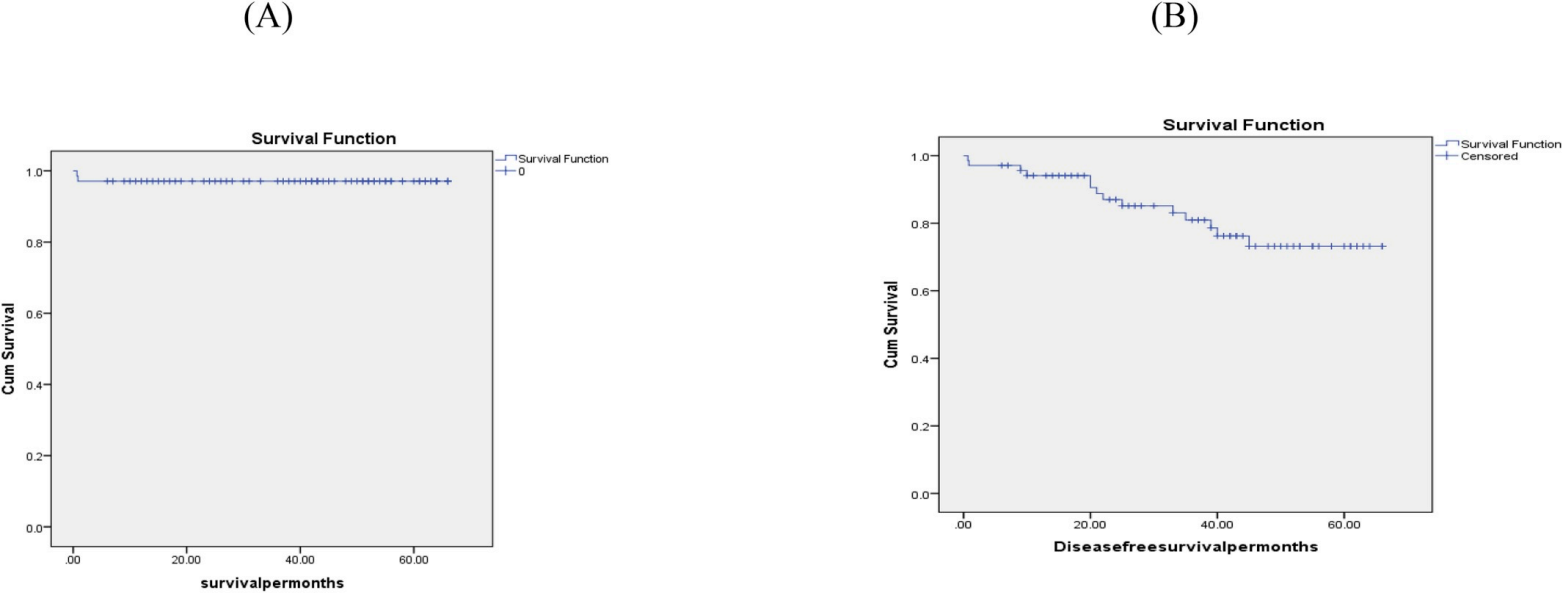
A- KM survival curve.

On the other hand, the late biliary morbidity was 12(17.4%), in the form of recurrent cholangitis 5(7.25%); where the initial attacks developed at 9 months, 10 months, 20 months, 33 months and 39 months from definitive surgery, stricture 5(7.25%), that occurred at 20 months, 21 months, 22 months, 25 months, and 35 months from surgery, and both stricture and recurrent cholangitis 2(2.9%), that happened at 40 months and 45 months from surgery; as regard McDonald's grading, grades A, B, C, and D were 45/67(67.2%),10/67(14.9%),5/67(7.5%), and 7/67(10.4%) respectively; these complications were managed as follow: Medical treatment for the 5 cases with recurrent cholangitis, PTD for 3 cases with stricture, and redo HJ for the 2 cases with both stricture and recurrent cholangitis and the other 2 cases with stricture only, moreover; all these patients improved after management. There was no other late mortality except the 2 cases with early mortality, however, the overall survival and the late biliary morbidity-free survival were 67(97.1%), and 55(79.7%) respectively. Table 3, Fig. 7.

**Table 3:** Outcome after the definitive procedure.

Character	NO	(%)
	69	(100%)
	(Mean ± SD)	
Early(Hospital and one month)morbidity	15	(21.7%)
Infection(pulmonary and/or wound)	10	(14.5%)
Abdominal collection	4	(5.8%)
Bile leak	4	(5.8%)
Cholangitis	4	(5.8%)
Liver failure	2	(2.9%)
Clavien grading of early complications(CDS)		
2	6	(8.7%)
3	6	(8.7%)
4	1	(1.4%)
5	2	(2.9%)
Early mortality	2	(2.9%)
Cause of death	Liver failure, Sepsis	
Hospital stay after definitive management (days) (Mean ± SD)	8.6 ± 5.2	
Late biliary morbidity	12	(17.4%)
Recurrent cholangitis	5	(7.25%)
Stricture	5	(7.25%)
Stricture, recurrent cholangitis	2	(2.9%)
McDonald's grades/67		
A	45	(67.2%)
B	10	(14.9%)
C	5	(7.5%)
D	7	(10.4%)
Management of late biliary morbidity		
Medical	5	(7.2%)
PTD	3	(4.3%)
Redo HJ	4	(5.8%)
Late mortality	2	(2.9%)
Overall survival	67	(97.1%)
Late biliary **morbidity-free** survival	55	(79.7%)
Survival(months) (Mean ± SD)	39.5 ± 19	
Late biliary **morbidity-free** survival(months) (Mean ± SD)	36.1 ± 18.7	

CDS: Clavien Dindo score, PTD: Percutaneous transhepatic drainage, HJ: Hepaticojejunostomy.

### 2.4 Predictors of early morbidity

On univariate analysis, the following factors were significant predictors of early morbidity: Ligature followed by clipping as mechanisms of injury, injury discovery in the intermediate period after cholecystectomy (between 1 week and 3 months), sepsis at referral, obstructing and vascular form of injury, higher Strasberg grade, associated vascular injury, intervention before definitive repair, performing the definitive surgery later after injury (mean 140 ± 60.4 days), RT hepatectomy with biliary reconstruction as the definitive procedure, operative bleeding and blood transfusion (mean 0.9 ± 1.4 units), cirrhotic liver during definitive procedure and longer mean operative time and hospital stay(296 ± 79.2 min and 16.6 ± 5.5 days respectively). On the other hand, there was a trend towards the occurrence of significant early morbidity with later referral after injury discovery (mean 10.8 ± 10.05 days). In contrast, there was no independent predictor of early morbidity on multivariate analysis. Table 4, Table 5.

**Table 4:** Predictors of early (hospital and 1 month) morbidity.

Category	Early morbidity		p-value Univariate analysis	P value Multivariate analysis
	Number	(%)		
Number of patients	15/69	(21.7%)		
Gender			>0.05	
Males	6/31	(19.4%)		
Females	9/38	(23.7%)		
Direct cause of injury			0.03	
Clipping	5/12	(41.7%)		
Diathermy	7/43	(16.3%)		
Ligature	3/6	(50%)		
Scissor	0/8	0		
Time of Injury diagnosis			0.049	>0.05
During cholecystectomy operation	1/14	(7.1%)		
Early before 1 week	5/31	(16.1%)		
Intermediate 1 week to 3 months	8/18	(44.4%)		
Late after 3 months	1/6	(16.7%)		
Main presentation			0.3	>0.05
During **cholecystectomy** operation	1/14	(7.1%)		
Jaundice	5/16	(31.3%)		
Cholangitis	4/12	(33.3%)		
bile from drain	3/21	(14.3%)		
Peritonitis	2/6	(33.3%)		
Sepsis at referral			0.019	>0.05
Yes	5/10	(50%)		
No	10/59	(16.9%)		
BI type			0.001	>0.05
Leaking	130	(3.3%)		
Obstructing	6/19	(31.6%)		
Both leaking and obstructing	3/13	(23.1%)		
Leaking and vascular	1/2	(50%)		
Obstructing and vascular	4/5	(80%)		
Strasberg classification of injury			0.000	>0.05
E1	2/25	(8%)		
E2	6/32	(18.8%)		
E3	3/8	(37.5%)		
E4	4/4	(100%)		
Associated vascular injury			0.001	>0.05
Yes	5/7	(71.4%)		
No	10/62	(16.1%)		
Intervention before definitive treatment			0.035	>0.05
Yes	15/56	(26.8)		
No	0/13	(0)		
Laparotomy prior to definitive repair			>0.05	
Yes	4/21	(19%)		
No	11/48	(22.9%)		
Time of definitive procedure from injury			0.1	>0.05
Immediate (before 72 h)	0/9	(0)		
Intermediate (between 72 h and 1.5 months)	1/10	(10%)		
Late (after1.5 months)	14/50	(28%)		
Definitive procedure			0.001	>0.05
End to end biliary anastomosis with stent	0/3	(0)		
HJ with stent	5/40	(12.5%)		
RT hepatectomy, HJ with stent	5/6	(83.3%)		
HJ **without stent**	5/20	(25%)		
Stenting			>0.05	
Yes	10/49	(20.4%)		
No	5/20	(25%)		
Operative bleeding			0.000	>0.05
Yes	9/10	(90%)		
No	6/59	(10.2%)		
Intra operative liver biopsy			0.000	>0.05
Cirrhotic	10/13	(76.9%)		
Normal	5/56	(8.9%)		

BI: Biliary injury, HJ: Hepaticojejunostomy.

**Table 5:** Predictors of early morbidity.

Category	Early morbidity (Mean ± Std. deviation)	No morbidity (Mean ± Std. deviation)	p-value Univariate analysis	p-value Multivariate analysis
Age	36.1 ± 9.9	38.6 ± 9.5	>0.05	
Referral time after injury diagnosis (days)	10.8 ± 10.05	6.1 ± 8.3	0.1	>0.05
Total bilirubin	6.2 ± 1.7	6.6 ± 7.1	>0.05	
Direct bilirubin	5.4 ± 1.9	5 ± 5.4	>0.05	
Time of definitive surgery after injury(days)	140 ± 60.4	56.3 ± 48.8	0.000	>0.05
Operative time(min)	296 ± 79.2	208.9 ± 77.8	0.001	>0.05
Blood transfusion(units)	0.9 ± 1.4	0.000 ± 0.000	0.000	>0.05
Hospital stay after definitive management (days)	16.6 ± 5.5	6.4 ± 1.9	0.000	>0.05

### 2.5 Predictors of late biliary morbidity

On univariate analysis, the following factors were significant predictor's of late biliary morbidity: Sepsis at referral, performing the definitive surgery later after injury (mean 114.8 ± 84.9 days), end to end anastomosis with stenting as the definitive procedure, the absence of stenting, operative bleeding, cirrhotic liver, and early morbidity. Conversely, on multivariate analysis, there was no independent risk factor of our late biliary morbidity. Table 6, Table 7.

**Table 6:** Predictors of late biliary morbidity.

Category	Late biliary morbidity		p-value univariate analysis	p-value mutivariate analysis
	Number	(%)		
Number of patients	12/69	(17.4%)		
Gender			0.1	>0.05
Males	3/31	(9.7%)		
Females	9/38	(23.7%)		
Direct cause of injury			0.1	>0.05
Clipping	5/12	(41.7%)		
Diathermy	5/43	(11.6%)		
Ligature	1/6	(16.7%)		
Scissor	1/8	(12.5%)		
Time of Injury diagnosis			>0.05	
During cholecystectomy operation	3/14	(21.4%)		
Early before 1 week	5/31	(16.1%)		
Intermediate 1 week to 3 months	4/18	(22.2%)		
Late after 3 months	0/6	(0)		
Main presentation			0.07	>0.05
During **cholecystectomy** operation	3/14	(21.4%)		
Jaundice	0/16	(0)		
Cholangitis	5/12	(41.7%)		
Bile from drain	3/21	(14.3%)		
Peritonitis	1/6	(16.7%)		
Sepsis at referral			0.003	>0.05
Yes	5/10	(50%)		
No	7/59	(11.9%)		
BI type			0.3	>0.05
Leaking	4/30	(13.3%)		
Obstructing	6/19	(31.6%)		
Both leaking and obstructing	2/13	(15.4%)		
Leaking and vascular	0/2	(0)		
Obstructing and vascular	0/5	,(0)		
Strasberg classification of injury			>0.05	
E1	4/25	(16%)		
E2	6/32	(18.8%)		
E3	2/8	(25%)		
E4	0/4	(0)		
Associated vascular injury			>0.05	
Yes	0/7	(0%)		
No	12/62	(19.4%)		
Intervention before definitive treatment			>0.05	
Yes	10/56	(17.9)		
No	2/13	(15.4%)		
Laparotomy prior to definitive repair			>0.05	
Yes	4/21	(19%)		
No	8/48	(16.7%)		
Time of definitive procedure from injury			>0.05	
Immediate (before 72 h)	2/9	(22.2%)		
Intermediate (between 72 h and 1.5 months)	1/10	(10%)		
Late (after1.5 months)	9/50	(18%)		
Definitive procedure			0.004	>0.05
End to end biliary anastomosis with stent	2/3	(66.7%)		
HJ with stent	3/40	(7.5%)		
Rt hepatectomy, HJ with stent	0/6	(0)		
HJ **without stent**	7/20	(35%)		
Stenting			0.014	>0.05
Yes	5/49	(10.2%)		
No	7/20	(35%)		
Operative bleeding			0.003	>0.05
Yes	5/10	(50%)		
No	7/59	(11.9%)		
Intra operative liver biopsy			0.000	>0.05
Cirrhotic	7/13	(53.8%)		
Normal	5/56	(8.9%)		
Hospital and 1 m morbidity			0.009	>0.05
Yes	6/15	(40%)		
No	6/54	(11.1%)		

BI: Biliary injury, HJ: Hepaticojejunostomy.

**Table 7:** Predictors of late biliary morbidity.

Category	Late biliary morbidity (Mean ± Std. deviation)	No Late biliary morbidity (Mean ± Std. deviation)	p-value univariate analysis	p-value Mutivariate analysis
Age	35.9 ± 8.7	38.5 ± 9.7	>0.05	
Referral time after diagnosis (days)	3.1 ± 3.8	7.9 ± 9.4	>0.05	
Total bilirubin	5.9 ± 6.3	6.6 ± 6.4	>0.05	
Direct bilirubin	4.7 ± 5.2	5.2 ± 4.9	>0.05	
Time of definitive surgery after injury(days)	114.8 ± 84.9	66.03 ± 52.9	0.01	>0.05
Operative time(min)	250 ± 86.1	223.2 ± 85.5	>0.05	
Blood transfusion(units)	0.08 ± 0.2	0.2 ± 0.8	>0.05	
Hospital stay after definitive management (days)	11.4 ± 6.4	7.9 ± 4.8	0.1	<0.05

## 3 Discussion

BDI is considered the most significant complication of LC [9,20,47]. The rate of MBDI (type E) of Strasberg's classification vary from 0.08 to 0.6% and is associated with significant morbidity and mortality [2,17,18,31,48,49]. Most of the patients with these injuries are referred to tertiary referral centers; either immediately or after unsuccessful re-operation [27,31,50]. Higher rates of successful repair have been reported in these referral centers [2,19,25,38,48,51,52]; so, it is highly recommended for patients to be referred early to these centers [18]. Similarly, as our center is a tertiary referral hepatobiliary one, 68 of the 69 patients were referred to us from other hospitals either before the definitive procedure (66 patients) or after it (2 patients) where our long-term success rate reached 82.6%.

A detailed preoperative evaluation by(Laboratory(I.e. LFT,…), and imaging(US, CT, MRCP, ERCP, PTC, fistulogram,….), improving of patient general condition before the definitive procedure by co-operation of surgeons, endoscopists and intervention radiologists, correction of nutritional, fluid-electrolyte disorders and controlling sepsis; and then finally performing meticulous wide anastomosis by experienced surgeons in specialized hepato-biliary units are required for achieving long-term success after repair of MBDIs [53]. Similarly, in the current cohort, after referral to our center, we performed abdominal US, CT abdomen, abdominal CT angiography, MRCP, PTC, and ERCP in 65(94.2%), 14 (20.3%), 11 (15.9%), 40(58%), 10(14.5%), and 35(50.6%) of our patients respectively to delineate biliary anatomy and to determine the type of injury. Furthermore, the 10 patients presented with sepsis at referral were properly managed to control their condition before the definitive procedure. Moreover, multidisciplinary staff meeting including surgeons, radiologists, endoscopists, and anaesthetists occurred for controlling initial patient condition where 81.2% of patients underwent the required intervention procedures (laparotomy, endoscopy and/or intervention radiology) before the definitive operation according to the staff meeting decision.

In the current cohort, we analyzed the early and late outcomes after the definitive procedures.

The surgical principles associated with a successful repair of MBDI are exposure of well-vascularized healthy proximal bile ducts that drain the entire liver, and preparation of a Roux-en-Y limb of jejunum>60 cm for a mucosa to-mucosa, tension-free anastomosis between them [2,15,53]. The Roux-en-Y HJ for MBDIs had the best early and late outcomes [5,29,30,[54], [55], [56], [57]]. In similar, we had acceptable early and long-term outcome after performing Roux-en-Y HJ (Hepp–Couinaud technique) bilio-enteric reconstruction where it leads to only 10/60(16.7%) early morbidity and 50/60(83.3%) good long-term biliary outcome (according to McDonald grading). Furthermore, it was our most frequent operation(87%). Similarly, The Roux-en-Y HJ offered good long-term outcome or success in 83%,88.3%, 89%, 90%,91.3%, 92%, 92%, 94%, and 97% of patients in Schmidt et al., 2004 [58], Schmidt et al., 2005 [21], De Reuver et al., 2007 [29], Pottakkat et al., 2010 [3], Lubikowski et al., 2011 [59], Mishra et al., 2015 [37], Bansal et al., 2015 [36], Benkabbou et al., 2013 [30], AbdelRafee et al., 2015 [5], studies respectively.

Hepatectomy as a management of MBDIs is associated with a high postoperative morbidity rate [20,30,[33], [34], [35],^53^]. In similar, the hepatectomy that was performed in 8.7% of our patients had a significant negative impact on early morbidity (83.3%, P > 0.05); also, Pekolj et al., in 2015 [20], Li et al., in 2012 [35], and Schmidt et al., in 2010 [60] reported 60%, 60%, and 50% morbidities respectively when it was used as a treatment of MBDIs. It is indicated with higher biliary injuries associated with vascular injuries, liver abscesses or atrophy and after multiple failed previous repairs [34,[60], [61], [62], [63]]; as liver resection removes the diseased biliary confluence and the atrophic segment providing good access to the remnant bile duct for a healthy safe anastomosis [33,34,64]. In the same way; these operations were done in our patients with grades E3, E4 associated with vascular injuries with the presence of liver abscesses and/or atrophy where proximal injuries and co-vascular injuries were significant predictors of hepatectomy (p = 0.00, and 0.00 respectively). Also, Proximal BDIs and injury to the right hepatic artery were independent risk factors of hepatectomy in Li et al., 2012 [35] and Truant et al., 2010 [64] studies.

Primary end-to-end repair of MBDI is a method of repair used when there is no loss of tissue [65]. When using this reconstruction method, the mobilization of the bile duct should be minimal to avoid devascularization and stricture development, however it is associated with a high failure rate [2,40]; this failure occurs due to destruction of the axial blood supply of the extrahepatic bile duct due to marked dissection leading to ischemia and repair failure [14]. It had a significant negative impact on our late biliary morbidity. Similarly, it was associated with 33.3% early morbidity and 33.3% late stricture in Perrakis et al., 2015 [17] study. Also, it was significantly associated with late stricture formation in Csendes et al., 1989 [66] study.

The use of trans-anastomotic stents is controversial [2,7,15,67]. However, some investigators reported the benefit of stents in avoiding recurrent cholangitis [68]. In the same line, we used trans-anastomotic stents in 49/69(70%) of our patients, these stents had a positive impact on our long-term biliary outcome; this was due to the adequate biliary drainage and flow through the anastomosis and the lower intraductal pressure with stents. Similarly, Laukkarinen et al., 2010 [69], found low rates of anastomotic leakage or stricture in their experimental models when performed Roux-en-Y HJ with a transanastomotic stents, also, Moris et al., 2016 [70] recorded low stricture rate when performed HJ with stenting for biliary obstruction of different causes. On contrary, the outcome of Roux-en-Y HJ that was performed laparoscopically by Cuendis-Vela´zquez et al., 2016 [7] was good at maximum 36 months follow-up without using stents.

The reported both early and late morbidity rates after repair of BIs ranged from 38% to 65% [5,17,24,50,71]. Despite the presence of little literature on the long-term outcomes after surgical reconstruction of MBDIs, the literature rates of long-term post-repair strictures were extremely variable between 4% and 38% [5,21,24,31,58,70]. However, in the current study, both early and late morbidities affected 30.4% of patients; this lower rate was due to the inclusion of only late biliary morbidity, not all late morbidities; these late biliary morbidities included recurrent cholangitis 7.25%, stricture 7.25%, and both stricture and recurrent cholangitis 2.9%.

Despite the presence of scarce literature on the factors affecting post repair stricture, some factors were mentioned, (I.e. Associated sepsis, high level of injury, vascular injury, timing of repair, operative technique, multiple prior attempts at repair, presence of hepatic parenchymal disease, portal hypertension, unavailability of a preoperative complete delineation of the injury by cholangiography, and surgeon's inexperience) [24,50,53,60]. In this cohort, we analyzed the factors affecting early morbidity as well as late biliary morbidity.

In our study, referral time to our center after injury diagnosis had no significant impact on early complications or late biliary morbidity; despite the trend towards occurrence of significant early complications with longer time, similarly, In a multivariate analysis done by Lillemoe et al., 2000 [72], the interval until admission to a tertiary hepatobiliary center had no significant impact on the outcome. In contrast, it independently affected short- and long-term outcomes in Bansal et al., 2015 [36] study and was independent predictor of worse outcome in De Reuver et al., 2007 [29] and Martinez-Lopez et al.,2017 [52] studies, also, longer delay of referral (>3 months) from index surgery was associated with poor outcome in AbdelRafee et al., 2015 [5] study.

Intra-abdominal sepsis and abscesses even if drained effectively may remain active in the period after surgery, predisposing patients to fibrosis, resulting in late anastomotic stricture. Furthermore, inflammatory changes in the surgical bed produce tissue friability, resulting in increased technical difficulty at repair time [38,73]. In similar, in our study, sepsis at referral due to biliary peritonitis or severe cholangitis was significant predictor of early and late morbidities, despite our aggressive management of it before doing the definitive repair, similarly, it was independent predictor of complications and anastomotic failure after primary repair in Dominguez-Rosado et al., 2016 [19] study and was predictor of severe complications in Patrono et al., 2015 [4] study and it was the only independent predictor of major morbidity and a significant predictor of late biliary stricture in Sulpice et al., 2014 [31] study, in the same line, it was independent predictor of long-term complications in Huang et al., 2014 [38] study. In the same way, Schmidt et al., 2005 [21] found that the presence of active peritonitis was independently associated with long-term complications, such as anastomotic stricture, or secondary biliary cirrhosis. Similarly, repair at a stage with active biliary or peritoneal inflammation was a significant predictor of long-term failure in Huang et al., 2003 [12] study. In contrast, it did not affect outcome after surgical repair of injury in Walsh et al., 2007 [24] and Lubikowski et al., 2011 [59] studies.

Repair in patients with higher strictures (Strasberg- Bismuth types III, IV, and V was a predictor of failure in some series [21,74]. However, in our study, it had no effect on late biliary outcome despite its effect on early complications. Also, it did not affect post repair long-term success rate in Pottakkat et al., 2010 [3] and Lubikowski et al., 2011 [59] studies. Conversely, it was independently associated with an overall poor short- and long-term outcomes in Bansal et al., 2015 [36] study, and was a significant predictor of postoperative stricture in Walsh et al., 2007 [24] study.

Because post-LC BIs occur more proximally in comparison to OC, a higher incidence of concomitant vascular injury can be anticipated [58,73]. Similarly, we found a significant correlation between the higher level of injury and co-vascular injuries (P = 0.000). The rate of concomitant RT-HA injury was variable between 7% and 40% in different series [2,8,18,30,31,52,59,[75], [76], [77]]; depending on the cohort of patients under evaluation and whether angiographic assessment was done routinely [53]. However, it was less (8.6%) in our work; and this lower rate may be due to the nonroutine performance of pre-operative CT angiography or intra-operative doppler us as they were performed only when vascular lesions were suspected.

Despite concomitant vascular injury was associated with early morbidity in our work, it had no effect on our late biliary morbidity, also, it had no effect on post-operative late biliary outcome in Walsh et al., 2007 [24], Sulpice et al., 2014 [31], Lubikowski et al., 2011 [59], Keleman et al., 2011 [78], and Pulitano et al., 2011 [79] studies. In contrast, Bachellier et al., 2001 [80], Koffron et al., 2001 [81], and Sarno et al., 2012 [82] found that patients with concomitant vascular injuries had long-term worse outcomes, and Bansal et al., 2015 [36] found an independent correlation between concomitant vascular injury and both short- and long-term outcomes, moreover, Schmidt et al., 2004 [58] found an independent correlation between concomitant vascular injuries and post-repair biliary complications, and Buell et al., 2002 [71] reported an increased morbidity rate, and worse long-term outcomes in patients presenting with simultaneous biliary and vascular injuries.

The optimal time of BI repair remains controversial, moreover, it is determined by the general condition of the patient, favorable local abdominal factors for successful repair (absence of inflammation, collections, and sepsis), and the surgeon experience [55]. Performing the definitive operation immediately (during the 1st 72 h), in the intermediate period (between 72 h and 1.5 months) or in the late period (after1.5 months) from injury, did not have any significant effect on our late biliary morbidity. Similarly, it did not influence postoperative morbidity in Pottakkat et al., 2010 [3], Patrono et al., 2015 [4], Bansal et al., 2015 [36], Huang et al., 2014 [38], Kirks et al., 2016 [83], and Perera et al., 2011 [84], studies. However, most authors advice late repair of LC-BDIs (≤6 weeks) from LC [29,53,85]; this allows the abdominal inflammation to subside prior to definitive repair leading to its success [50,86]. Furthermore, it allows the biliary anatomy to be defined and the bile duct ischemic damage to be determined [20]. The long-term outcome after late repair was good in Chapman et al., 1995 [74], Murr et al., 1999 [87], and Sikora et al., 2006 [88] studies.

In another line, Arora et al., 2015 [89] found excellent long-term outcomes with immediate repair (>72 h). On the other hand, several authors reported a higher rate of postoperative biliary stricture when repairs were performed in the early period (>6weeks) from LC [13,24,29,79,90].

Chronic extra-hepatic biliary obstruction results in dilatation of the extra and intrahepatic biliary tree and proliferation of biliary ductules within the portal triads leading to fibrosis of portal tracts that results in secondary biliary cirrhosis and portal hypertension [14,51,54]. The onset of secondary biliary cirrhosis is variable and likely depends on many factors, such as degree and duration of symptomatic obstruction, occurrence, and frequency of cholangitis, and long interval between cholecystectomy and HJ [14,33,53,88]; so, in case of late referral, patients should be carefully assessed for signs of portal hypertension and secondary biliary cirrhosis [48]. However, 2ry biliary cirrhosis may occur as early as 20 weeks from the time of MBDI [91]. The median interval from bile duct injury to the evolvement of secondary biliary cirrhosis was 8 months in Schmidt et al., 2010 [60] study; owing to the rapid progress from recurrent cholangitis to secondary biliary cirrhosis. In similar, we found that performing the definitive surgery later after injury (mean 140 ± 60.4 days) had a significant negative impact on our early morbidity, and performing it at a mean of 114.8 ± 84.9 days had a significant negative impact on our late biliary morbidity. This may be explained by the development of biliary cirrhosis that had a significant negative impact on both early and late outcomes when the repair was so delayed in some of our cases. Similarly, cirrhosis was an independent predictor of post-surgical repair biliary stricture in Sulpice et al., 2014 [31] study, and also, cirrhosis and portal hypertension were independent predictors of failure of repair in Pottakkat et al., 2010 [3] study.

To the best of our knowledge, previous literature did not mention any correlation between operative bleeding and the occurrence of early or late morbidities after surgical repair of MBDIs, however, we found a significant correlation between intra-operative bleeding and both early and late morbidities. Our explanation is that bleeding was related to cases that underwent hepatectomy (P = 0.00) that had a negative impact on early morbidity, and also, was associated with cases with liver cirrhosis (P = 0.00) that had a negative impact on both early and late morbidities.

The occurrence of major postoperative complications (CDS>3) were associated with an increased risk of biliary stricture after surgery in Sulpice et al., 2014 [31] and Booij et al., 2018 [92] studies, in the same line, early morbidity was significant predictor of late biliary morbidity in our cohort, and it was independent predictor of late stricture in AbdelRafee et al., 2015 [5] study. In conclusion, Sepsis at referral, liver cirrhosis, and operative bleeding were significantly associated with both early and late morbidities after definitive management of LC related MBDIs, so it is crucial to avoid these catastrophes when doing those major procedures.

The main limitation of the study is being retrospective with relatively small NO of patients. So, it is advisable to do further studies with larger no and longer follow-up period with stress on the effect of blood transfusion on morbidities.

## Ethical approval

The approval by National liver institute, Menoufiya university.

## Sources of funding

No source of funding for this research.

## Author contribution

Emad Hamdy Gad: Study design, data collection, writing, statistical analysis and publication.

Eslam Ayoup: Reference update.

Yasmin Kamel: data collection, writing, statistical analysis.

Talat Zakareya: data collection, writing.

Mohamed Abbasy: data collection, writing.

Ali Nada: data collection, writing.

Mohamed Housseni: data collection, writing.

Mohammed Al-sayed Abd-elsamee: Reference update.

## Conflicts of interest

No conflict of interest to declare.

## Research registry number

researchregistry2211.

## Guarantor

All the authors of this paper accept full responsibility for the work and/or the conduct of the study, had access to the data, and controlled the decision to publish.

### Provenance and peer review

Not commissioned externally peer reviewed.

## Appendix A Supplementary data

checklist checklist

checklist checklist
